# Photocatalytic Oxidation of VOCs in Gas Phase Using Capillary Microreactors with Commercial TiO_2_ (P25) Fillings

**DOI:** 10.3390/ma11071149

**Published:** 2018-07-06

**Authors:** Javier Fernández-Catalá, Ángel Berenguer-Murcia, Diego Cazorla-Amorós

**Affiliations:** Inorganic Chemistry Department, Materials Institute, University of Alicante, Ap. 99, E-03080 Alicante, Spain; j.fernandezcatala@ua.es (J.F.-C.); a.berenguer@ua.es (Á.B.-M.)

**Keywords:** TiO_2_, microreactor, photocatalytic activity, VOCs, propene

## Abstract

The elimination of volatile organic compounds (VOCs) at low concentration is a subject of great interest because these compounds are very harmful for the environment and human health. In this work, we have developed an easy methodology to immobilize a benchmark photocatalyst (P25) inside a capillary microreactor (Fused silica capillary with UV transparent coating) without any previous treatment. For this purpose, a dispersion of the sample (P25) in EtOH was used obtaining a packed bed configuration. We have improved the immobilization of the benchmark photocatalyst (P25) inside the capillary incorporating a surfactant (F-127) to generate porosity inside the microreactor to avoid severe pressure drops (∆P < 0.5 bar). The resulting capillaries were characterized by Scanning Electron Microscopy (SEM). These microreactors show a good performance in the abatement of propene (VOC) under flow conditions per mol of active phase (P25) due to an improved mass transfer when the photocatalyst is inside the capillary. Moreover, the prepared microreactors present a higher CO_2_ production rate (mole CO_2_/(mole P25·s)) with respect to the same TiO_2_ operating in a conventional reactor. The microreactor with low pressure drop is very interesting for the abatement of the VOCs since it improves the photoactivity of P25 per mol of TiO_2_ operating at near atmospheric pressure.

## 1. Introduction

The removal of volatile organic compounds (VOCs) at low concentrations is a hot topic nowadays because these compounds are very harmful to the environment and human health. These pollutants present carcinogenic effects and damage the central nervous system and are directly related to climate change, among several other adverse effects [1,2,3]. Propene removal at low concentrations is interesting because this molecule might be taken as a representative example of low molecular weight VOCs [4,5]. Heterogeneous PhotoCatalytic Oxidation (PCO) is a novel way to eliminate this type of pollutant because it can be performed at room temperature and at atmospheric pressures [6]. Titania (TiO_2_) has received significant attention for this purpose due to its unique properties, including photocatalytic activity, photo- and chemical stability, nontoxicity, and relatively low production cost [7,8,9]. An interesting commercial TiO_2_ material is P25 (Evonik, Essen, Germany), which has established itself as the benchmark for photocatalysis applications. This titanium dioxide-based solid consists of 80% anatase and 20% rutile [10]. This commercial titania nanopowder is widely used as a catalyst in photochemical reactions due to its very high photocatalytic activity [11].

An interesting approach for the efficient elimination of propene would be the use of photomicroreactor technology. In recent decades, the use of microreactor technology has experienced a great increase in diverse fields of scientific disciplines such as medicine [12], chemistry [13,14,15], materials science [16,17], and energy [18], among others. In the field of heterogeneous catalysis, the use of these devices has focused primarily in organic synthesis [19] and abatement of contaminants in microfluidic reactors [20] mainly due to the benefits of microreactors with respect to conventional reactors. These include large surface-to-volume ratio, higher mixing efficiency, faster heat and mass transfer rates, shorter reaction time and higher reaction rate [16,19,21]. Concerning photochemistry, another important factor may be the enhanced efficiency and homogeneity in light irradiation in the microreactor due to the small dimensions characteristic of these devices. This in turn allows the use of lighting sources which consume less power such as LEDs [19].

At present, different types of microreactors are employed for heterogeneous catalysis, such as packed-bed, monolithic and wall-coated microreactors [22]. The former, composed by packed microparticles of a solid catalyst inside the microchannel, is very interesting for solid-liquid reactions, due to a highly improved micromixing performance compared with non-packed microchannels [23], and for catalytic reactions in gas phase since it assures a more efficient contact with the catalyst [24]. However, the packed-bed microreactor strategy presents as the main drawback the immobilization of the catalyst inside the capillary, since the synthesis in-situ of the catalyst filling is not straightforward and may require synthesis conditions which are not compatible with the microreactor materials. In addition, the synthesis conditions have to be fine-tuned to get adequate interparticle space that assures low pressure drops. Thus, development of easy strategies to load the specific catalyst composition inside the microchannels is highly desirable.

This work reports a straightforward methodology for the incorporation of a benchmark TiO_2_-based photocatalyst (Evonik P25) inside a capillary microreactor (Fused silica capillary with UV transparent coating) without previous treatment using a dispersion of the active sample in EtOH with and without the use of surfactants. This photomicroreactor was tested in the removal of propene at low concentrations in the gas phase obtaining an improvement of the net photocatalytic activity (CO_2_ production rate) with respect to the photoactivity of the P25 in a conventional reactor. The capillary prepared with P25 and a surfactant (Pluronic F-127), with the purpose of creating porosity among the particles of P25 incorporated inside the microreactor, resulted in a photomicroreactor having a lower pressure drop (0.5 bars) than the microreactor filled with P25 only (4 bars), which is a substantial practical improvement. This photomicroreactor was tested in the removal of propene at low concentrations in the gas phase obtaining an improved photocatalytic activity (CO_2_ production rate) per mole of active phase than the microreactor filled with P25 only.

## 2. Materials and Methods

### 2.1. Materials

Fused silica capillaries with a UV transparent coating (i.d. 0.1 mm) and a length of 25 cm were purchased from PHOTON LINES (Madrid, Spain) and used as capillary microreactors.

The reactants utilized in this work without further purification were commercial titanium oxide (P25, Evonik) absolute ethanol (EtOH, 99.8%, Fisher Scientific, Hampton, NH, USA), Pluronic F-127 (F-127, Sigma-Aldrich, St. Louis, MO, USA), and deionized water.

### 2.2. Incorporation of P25 Inside Silica Capillary

The incorporation of a benchmark TiO_2_ sample such as P25 without treatment inside the fused silica capillaries was carried out by using a dispersion of the P25 in EtOH. This approach is a straightforward methodology to incorporate already synthesized catalysts compared to the methodology of synthesizing the catalyst inside the microreactor in packed-bed, monolithic or wall-coated configuration, which is the usual process reported in the literature [13,23,24].

As an illustrative example, the incorporation of P25 previously synthesized inside the capillary was performed as follows: 100 mg of titanium oxide (P25) were weighed and dispersed in 600 mg of EtOH. This dispersion was stirred vigorously for 30 min using a magnetic stirrer. The dispersion was injected into the capillary using a syringe (1 mL of capacity). The filled capillary was sealed and set on a device which rotated the capillary around its axis for 6 h. The sealed capillary was then annealed at 120 °C for 3 h. After annealing, the capillary was opened and dried overnight at 120 °C. This capillary was labelled Cap. P25.

We also prepared a P25-filled capillary creating porosity among the active particles inside the microreactor by incorporating the surfactant Pluronic F-127 in the dispersion. This capillary was prepared as follows: 20 mg of F-127 (16% wt. with respect to the mass of P25) was dissolved in 600 mg of EtOH (solution A). Then, 100 mg of the titanium oxide (P25) were weighed and dispersed in solution A. This dispersion was stirred vigorously for 30 min. The dispersion was injected into the capillary using a syringe (1 mL of capacity). The filled capillary was sealed and set on a device which rotated the capillary around its axis for 6 h. The sealed capillary was then annealed at 120 for 3 h. After annealing, the capillary was opened and dried overnight at 120 °C. The filled capillary was washed with cold water at 0 °C for 3 days, with the purpose of removing the surfactant F-127 and generate porosity among the particles of P25 inside the microcapillary. This sample was labelled Cap. P25/F-127.

### 2.3. Sample Characterization

Optical microscopy and Scanning Electron Microscopy techniques were employed for the characterization of the TiO_2_ (P25) inside the fused silica capillaries. The fillings (P25) of the capillaries were observed by a stereo microscope (EZ4 HD, Leica, Wetzlar, Germany). The morphology of the particles inside the capillaries was analyzed by scanning electron microscopy (JSM-840, JEOL, Akishima, Japan). In addition, in this work a micro balance (ME Micro Balance ME36S, Sartorius, Goettingen, Germany) with a weight resolution of up to 0.0001 mg was used for calculating the mass (mg) of P25 incorporated inside the microcapillaries from the difference in weight of the filled and empty capillaries.

### 2.4. Catalytic Tests.

The photocatalytic performance of the P25 incorporated in the fused silica capillaries (microreactor) or in a conventional reactor (prepared for comparison purposes) were studied using two different experimental systems designed in our laboratory.

**Figure 1:**
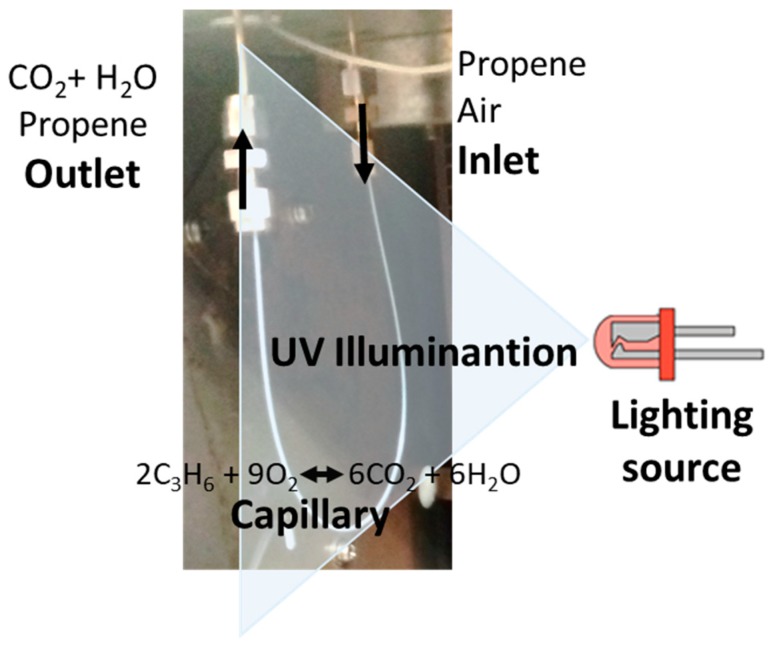
Illustration of the experimental set up used for photocatalytic oxidation of VOCs.

The system for the analysis of the catalytic activity of P25 incorporated inside fused silica capillary microreactors for the abatement of propene consisted in connecting the capillary in a GC chromatograph (GC-2010, Shimadzu, Kyoto, Japan). With the finality of studying the possible effect of the better illumination efficiency in microreactor systems, different types of UV light source were used. On the one hand, one UV LED or 6 UV LED lights (model: NSSU123T, Nichia Corporation, Anan, Japan) were used (the power of one LED is 0.015 W and the radiation peak appears at 375 nm). A factor to be taken into account in photocatalysis is the illumination (radiation power) reaching the photocatalyst. Since illumination depends on the distance from the LED to the sample as described by the Inverse-square law, we have used this law to get an estimate of the illumination of UV radiation reaching the photocatalyst. With the capillary being placed at a distance of 4 cm from the LEDs, the illumination decreased approximately by a factor of 16 using this configuration. On the other hand, a commercial UV lamp (Philips, Amsterdam, The Netherlands) with a power of 1 W with its radiation peak appearing at 365 nm, was also employed. This lamp is the one used for the conventional reactor and the illumination using this configuration is unaffected due to the proximity between the light source and the reactor (1 cm). A scheme of the system for capillaries is presented in Figure 1.

The microreactors prepared in this work (Cap. P25 and Cap. P25/F-127) were used for the oxidation of propene at 100 ppmv in air at room temperature, 25 °C. The calibrated gas cylinder was supplied by Carburos Metálicos, S.A. (Barcelona, Spain). The propene-containing stream had 100 ppmv of propene diluted in synthetic air and without the addition of any humidity. The flow rate of the propene-containing stream was 0.35 (STP) mL/min for the Cap. P25 and 1 (STP) mL/min for the Cap. P25/F-127 and the mass of P25 used in this study was 0.116 mg for Cap. P25 and 0.103 mg for Cap. P25/F-127 (Table 1). The difference in flow was caused by the high pressure drop of the former. In this work, we also studied the performance of the P25 in a conventional reactor using a system previously described [4]. The flow rate of the propene-containing stream was 30 (STP) mL/min and the mass of P25 used is 0.11 g in the experiment as shown in Table 1 [4,5].

The propene-containing stream was passed through the capillary or conventional reactor with photocatalyst. The outlet was injected into a GC chromatograph (6890N Agilent, Santa Clara, CA, USA) equipped with a CTR-I column (Alltech, Sabadell, Barcelona, Spain) operating at 30 °C. GC chromatography permits to follow the evolution of the concentration of propene in the outlet gas. Propene conversion was calculated using the following expression:

(1) $$\mathrm{Propene}\;\mathrm{conversion}\;\left(\%\right)=\frac{C_{initial_{C_{3}H_{6}}}-C_{steady_{C_{3}H_{6}}}}{C_{initial_{C_{3}H_{6}}}}\times 100$$

Where $C_{initial_{C_{3}H_{6}}}$ is the initial propene concentration, 100 ppmv, and $C_{steady_{C_{3}H_{6}}}$ is the propene concentration at steady state conditions when the UV light is switched on. Moreover, the CO_2_ production rate was calculated per mol of P25 using the following expression (with the aim to normalize the results with respect to the amount of P25):

(2) $${\mathrm{CO}}_{2}\;\mathrm{production}\;\mathrm{rate}\;=\;\frac{q_{gen}}{n}$$

where *q_gen_* is the molar flow rate of CO_2_ generated (moles CO_2_/s) and *n* is the moles of catalyst (moles of P25). The mass balances were checked in all reactions to corroborate that CO_2_ was the only reaction product, thus corroborating the selectivity of the photocatalytic process.

Additionally, blank tests were performed under the same experimental conditions as the catalytic tests but in the absence of the TiO_2_ photocatalysts. No catalytic activity was detected in any of the two reactor configurations.

## 3. Results and Discussion

### 3.1. Study of the Capillary Filled with P25 (Cap. P25)

In this section, we present the characterization results obtained with the capillary filled with a dispersion of P25 in EtOH as described in the Materials and Methods section. We also present and discuss the photocatalytic activity results comparing the results obtained with microreactors (Cap. P25) with respect to those obtained in a conventional reactor.

#### 3.1.1. Characterization of the Capillary Filled with P25 (Cap. P25)

**Figure 2:**
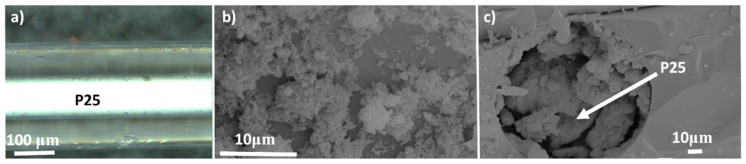
Microscopy images of the capillary sample Cap. 25 prepared in this study (**a**). (**b**,**c**) present the SEM images of the P25 inside the microcapillary for the same sample.

The general top view of the P25 filling inside the microcapillary is presented in Figure 2a. The optical microscope image shows a homogenous filling of the particles within the microcapillary of 100 μm in diameter. This figure shows an adequate filling and immobilization of the P25 particles inside the capillary with this methodology without any previous treatment. This procedure results in homogeneous P25 fillings inside 25 cm-long capillaries. However, this homogenous filling can block the capillary and produce high pressure drops, as will be presented below. Another proof of the filling of P25 particles inside the microcapillaries is observed in the SEM images obtained from the Cap. 25 sample as shown in Figure 2b,c.

#### 3.1.2. Photocatalytic Activity of the Capillary Filled with P25 (Cap. P25)

The photocatalytic activity of the capillary filled with P25 (Cap. P25) prepared in this work and the same active phase used in a conventional reactor were evaluated separately for comparison purposes. We studied the mineralization of propene in the gas phase at low concentration (100 ppmv in air) to CO_2_ at room temperature, following the global reaction showed in Equation (1) and as commented on in the Catalytic Test section.
2C_3_H_6_ + 9O_2_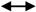 6CO_2_ + 6H_2_O(3)

It must be noted that in this study, we have not studied the mechanism of total photooxidation of VOCs since it has been extensively studied and reported in the literature [4,25]. Given that our samples do not differ significantly from those studies, it is safe to assume that the mechanism will be identical to that previously reported

**Figure 3:**
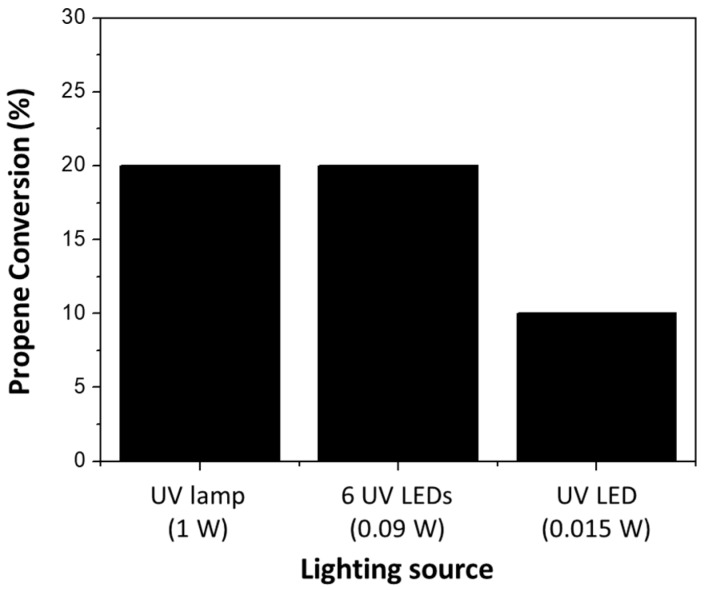
Study of the effect of the light source power in propene conversion using a UV lamp, 6 UV LEDs and 1 UV LED with 1W, 0.09 W and 0.015 W of power, respectively as light source.

With the purpose of studying the effect of the illumination efficiency in the capillary microreactor, the conversion of propene using the microcapillary filled with P25 was measured using UV light sources with different power outputs as mentioned in the Catalytic Test section (Figure 3). The results show that the 6 UV LEDs (with a total power of 0.09 W) and the 1 W UV lamp (normally used in conventional reactors in our previous studies [4]), produce very similar conversion levels. However, the conversion of propene decreased when only 1 UV LED (with a power of 0.015 W) was used.

**Figure 4:**
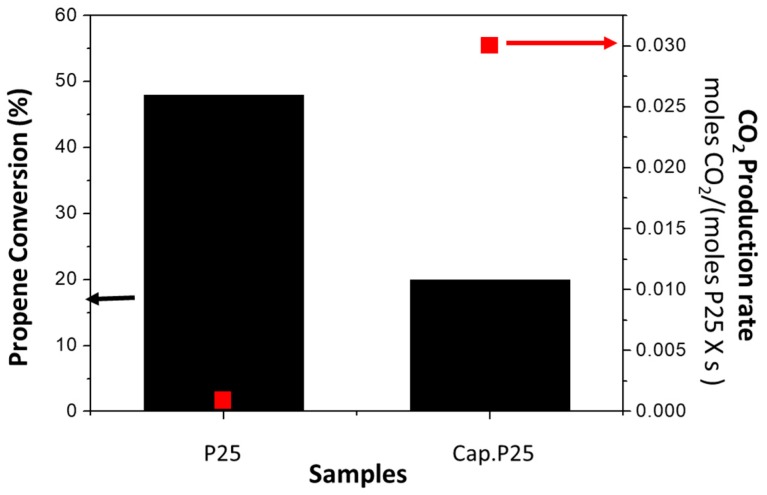
Comparison of propene conversion and normalized CO_2_ production rates for P25 incorporated in a conventional reactor (P25), and for sample Cap. P25.

With the finality of better understanding, the differences between the use of microcapillaries and conventional reactors, we compared the propene conversion in both reactor configurations as shown in Figure 4. The P25 powder placed in a conventional reactor presents a significantly higher value of propene conversion with respect to the P25 incorporated inside the microcapillary in absolute terms. This is in agreement with the higher residence time under the experimental conditions used in the conventional reactor (see Table 1). The sample Cap. P25 and the sample P25 in a conventional reactor present a residence time of 0.33 mg·min/mL and 3.66 mg·min/mL respectively, the residence time of sample Cap. P25 being one order of magnitude lower than the P25 placed in the convectional reactor. However, if we compare the CO_2_ production rate per mole of active phase (Figure 4), the capillary outperforms the conventional reactor. The CO_2_ production rate per mole of P25 in the Cap. P25 sample illuminated with 6 UV LEDS lights (0.009 W of power) is approximately 30 times (0.0305 moles CO_2_/(mols P25·s) greater than the conventional reactor illuminated with a commercial UV (Philips) light (1 W of power) (0.0009 moles CO_2_/(mols P25·s). Nevertheless, it must be noted that in these calculations we did not consider the different pressures in the conventional reactor and the microreactor and in this case the sample Cap. P25 presents a pressure drop of 4.5 bars (Table 1), which is a usual condition in this configuration [16,21].

In this work, we also tested the durability of the sample Cap. P25 in the conditions described in the Catalytic Test section, but we ran the propene abatement tests continuously for 12 h. The propene conversion decreased 3% after 12 h of reaction. This result is interesting because the sample Cap. P25 can operate in the propene conversion reaction in a stable way. No mass loss was observed inside of the capillary, indicating a good stability of the P25 filling.

### 3.2. Study of the Capillary Filled with P25 and F-127(Cap. P25/F-127)

The characterization results obtained with the capillary filled with a dispersion of P25, EtOH and F-127 as described above in the Materials and Methods section are presented in this section. This approach was carried out with the aim of overcoming the issues arising from high pressure drops (see above). By removing the surfactant (F-127), porosity is generated among the P25 particles inside the capillary which reduces the pressure drop (see Table 1). In this section, we have also studied and discussed the photocatalytic activity results obtained using this new approach compared with those of Cap. P25 and P25 incorporated in a conventional reactor.

#### 3.2.1. Characterization of the Capillary Filled with P25 and F-127(Cap. P25/F-127)

**Figure 5:**
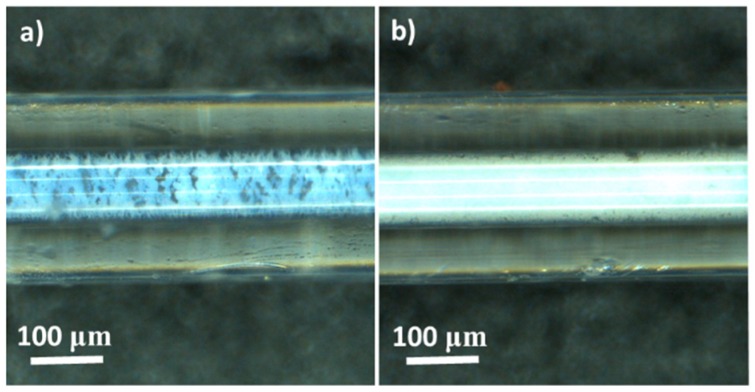
Microscope images of the different filled capillaries prepared in this study: (**a**) Cap. P25/F-127; (**b**) Cap. P25.

The optical microscopy images for Cap. P25/F-127 and Cap. P25 samples are included in Figure 5. The Cap. P25/F-127 sample presents less P25 inside the microcapillary with respect to the Cap. P25 sample. This result was also corroborated by weighing using a microbalance (see Table 1). Moreover, Figure 5 shows that sample Cap. P25/F-127 has a more heterogeneous filling due to interparticle spacing of the P25 produced by the use of the surfactant. This photocatalyst has a low pressure drop (0.5 bar), which is similar to the P25 powder in the conventional reactor. This result is interesting from an application point of view and for comparison purposes.

#### 3.2.2. Photocatalytic Activity of the Capillary Filled with P25 Using Surfactant (Cap. P25/F-127)

**Figure 6:**
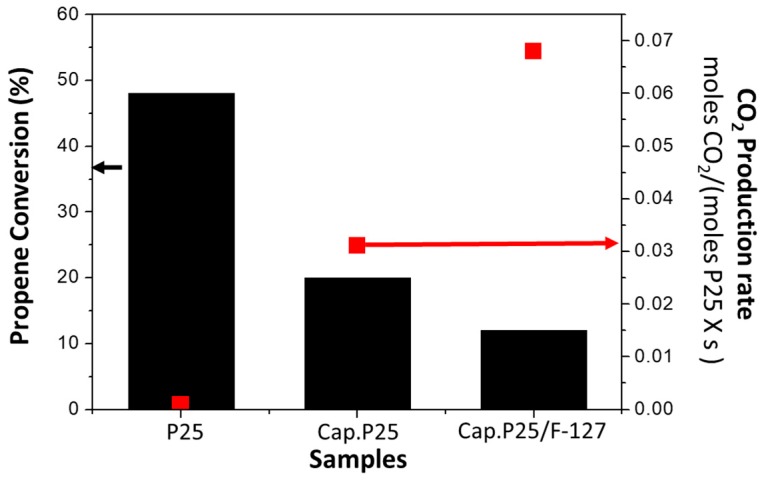
Comparison of propene conversion and CO_2_ production rates for P25 incorporated in a conventional reactor (P25), P25 incorporated in a microcapillary (Cap. P25) and P25 with surfactant incorporated in a microcapillary (Cap. P25/F-127).

Figure 6 includes the comparison in propene conversion for samples Cap. P25/F-127, Cap. P25 and P25 in a conventional reactor configuration. As already shown and discussed previously, the P25 in a conventional reactor presents the best value of propene conversion in absolute terms with respect to P25 incorporated inside the microcapillaries (Cap. P25 and Cap. P25/F-127). However, the conversion for the conventional reactor with respect to the capillary with comparable pressure drops is less than five times higher in spite of the fact that the residence time is more than 30 times higher. Interestingly, if we compare the CO_2_ production rate per mole of P25 (Figure 6), the Cap. P25/F-127 sample presents a significantly larger value than the other samples, being more than one order of magnitude (0.0685 moles CO_2_/(moles P25·s)) higher than for the conventional reactor (0.0009 moles CO_2_/(moles P25·s)). Comparing only the microcapillaries, it is observed that the CO_2_ production rate per mole of P25 in the Cap. P25/F-127 sample is approximately double (0.0685 moles CO_2_/(moles P25·s)) that of the Cap. P25 sample (0.0305 moles CO_2_/(mols P25·s)), even though the residence time is lower when the surfactant is used.

Considering the results obtained in this study in terms of characterization of the microcapillaries and their performance in the photocatalytic oxidation of propene at low concentrations, the most promising outlook seems to be the following: (1) we have established two different strategies for filling microcapillaries using a suspension of benchmark P25: one with ethanol and P25 alone and another with the same reagents but using a surfactant with the aim of creating porosity among the particles of P25; (2) Both microcapillaries (Cap. P25 and Cap. P25/F-127) present an improvement of the photoactivity per mole of photocatalyst probably due to an improved mass transfer. (3) The microcapillary with a porous packed bed presents a significant improvement in the photocatalytic activity per mole of P25 and a low pressure drop with respect to the microcapillary filled only with P25, making this capillary (Cap. P25/F-127) interesting for the elimination of VOCs in the gas phase.

Our results clearly show that it is possible to fill a microcapillary with the benchmark P25 photocatalyst in a packed bed configuration by a simple and cost-effective method. The prepared capillary microreactors display a remarkable photocatalytic activity with respect to the benchmark P25 in a conventional reactor. This approach opens the door to implementing microreactors with a photocatalyst inside (microphotoreactors) with high performance for the elimination of VOCs at low concentration with low pressure drops.

## 4. Conclusions

This work presents an easy methodology to immobilize a benchmark photocatalyst (P25) inside a capillary microreactor (Fused silica capillary with UV transparent coating), using a packed bed configuration without previous treatment, by using a dispersion of the sample (P25) in EtOH. This procedure has been modified using a non-ionic surfactant (F-127) in solution in order to create porosity inside the packed-bed inside the microreactor. In both cases the microreactors present a remarkable improved photocatalytic activity per mole of active phase (P25) with respect to the benchmark P25 disposed in the conventional reactor due to the improved mass transfer in the microreactor configuration. Moreover, the microcapillary with some porosity among the P25 particles presents the best photocatalytic performance expressed per mole of active phase (P25) and a pressure drop comparable to the conventional reactor used with a thin P25 bed. This approach opens up possibilities of applying this microtechnology with photocatalysts to the abatement of VOCs at low concentration in gas phase by overcoming one of its greatest drawbacks, which is the pressure drop across the reactor.

## Figures and Tables

**Table 1:** Mass of P25 inside the microreactors and the conventional reactor, flow rate of propene and the residence time used in the photooxidation experiments and registered pressure drop.

Samples	Mass of P25 (mg)	Propene Flux (mL/min)	Residence Time (mg·min/mL)	Pressure Drop (bar)
Cap. P25	0.116	0.35	0.33	4.5
Cap. P25/F-127	0.103	1	0.103	0.5
P25 in conventional reactor	110	30	3.66	0
